# Evaluating the Benefits of Collaboration in Simulation Games: The Case of Health Care

**DOI:** 10.2196/games.3178

**Published:** 2014-01-28

**Authors:** Ricky Leung

**Affiliations:** ^1^School of Public HealthDepartment of Health Policy, Management & BehaviorSUNY-AlbanyRensselaer, NYUnited States

**Keywords:** simulation games, health policy, social networks

## Abstract

**Background:**

Organizations have used simulation games for health promotion and communication. To evaluate how simulation games can foster collaboration among stakeholders, this paper develops two social network measures.

**Objective:**

The paper aims to initiate two specific measures that facilitate organizations and researchers to evaluate the effectiveness of Web-based simulation games in fostering collaboration.

**Methods:**

The two measures are: (1) network density and (2) network diversity. They measure the level of connectedness and communication evenness within social networks. To illustrate how these measures may be used, a hypothetical game about health policy is outlined.

**Results:**

Web-based games can serve as an effective platform to engage stakeholders because interaction among them is quite convenient. Yet, systematic evaluation and planning are necessary to realize the benefits of these games. The paper suggests directions for testing how the social network dimension of Web-based games can augment individual-level benefits that stakeholders can obtain from playing simulation games.

**Conclusions:**

While this paper focuses on measuring the structural properties of social networks in Web-based games, further research should focus more attention on the appropriateness of game contents. In addition, empirical research should cover different geographical areas, such as East Asian countries where video games are very popular.

## Introduction

### Study Design

Research has shown that games have great utility for health promotion and communication. Children with chronic health conditions can learn how to manage their diet and maintain healthy lifestyles with the aid of video games [1]; whereas elderly patients can increase reaction times and improve other cognitive tasks upon playing an appropriate amount of video games [2]. More recent research has also shown that physicians benefit from virtual games in receiving professional training [3].

Health organizations have started to use games for various pedagogical and communication purposes [1,3]. Two features of Web-based simulation games are important. First, these games enable players to experience collaboration. This experience is useful for players to better understand the benefits and difficulties in collaboration. Second, playing online is convenient. It allows players to connect without traveling, and game logistics can be handled by the computer.

Yet, these benefits need to be better evaluated to facilitate health organizations in applying games more systematically. In particular, while the individual-level benefits of games have received more attention in literature, it is unclear how simulation games foster collaboration. This paper develops two measures from the social network literature, network density (DEN) [4] and network diversity (DIV) [5], which can be used to evaluate the structure of collaboration in Web-based simulation games.

I first discuss selected findings in the relevant literature. This section provides a general orientation for the rest of this paper and also identifies research gaps that motivate my analysis. Next, I use a specific US health policy, the Patient Protection and Affordable Care Act (PPACA or ACA for short), to outline the roles of a possible Web-based simulation game. This outline will help illustrate how social networks in games can augment the individual benefits, and how the two proposed measures can be used empirically. In the final section, I summarize the paper’s major contributions and provide directions for further research.

### Relevant Studies

As mentioned, simulation games enable players to better understand specific issues with simulated experience [3,6-8]. Games that are endorsed and managed by a credible organization, such as an educational institute, a government agency, or an academic medical center, can be particularly effective in recruiting players and sustained participation. For example, the game “President for a Day”, developed by the Public Broadcast Service, seeks to engage school-aged children, and help them better understand the everyday life of US presidents. Assuming the role of a US president in the game, the player is given some freedom about possible actions, including attendance of different meetings in the White House, going to public hall hearings, delivering a speech, and so on [9].

In terms of knowledge-based benefits, simulated experience is able to improve rote memorization and traditional classroom learning. In one study, nurse training was shown to improve by up to 18% with simulation games [10]; in another study, surgeons could work 27% faster in laparoscopic surgery with simulation training [11]. In terms of affection-based benefits, research has shown that students of different ages have become more enthusiastic in national policies due to the simulated experience of a video game [12].

To evaluate individual-level benefits, whether knowledge- or affected-based, a health organization may examine whether the simulation game has increased the knowledge and affection of a targeted audience (eg, students or patients of certain groups) with a pre/post-test design or other quasi-experimental techniques [13]. Yet, these techniques are less effective in studying the collaboration among players. For health organizations to consider using games, it is useful to assess how players communicate and collaborate while playing the game, and whether these collaborations actually produce achievement.

Some researchers have already adopted indicators to measure the dissemination effectiveness of Internet contents. Open-source and commercial software is available to handle certain analytic tasks automatically. For example, an organization may build a Facebook company page to engage its stakeholders. The level of engagement may be captured by software [14] and measured by such indicators as the number of subscribers, the amount of discussions, and the frequency of share. The researcher or practitioner may also identify predictors, including organizational profile, geographical location, and contents of wall posts to assess their impact on various engagement indicators [15].

Yet, these indicators have shortcomings. Most importantly, since the number of users in specific Internet platforms varies, a sheer count on the number of feedback is insufficient. For example, in a social media platform with relatively few subscribers, a post that could generate feedback from subscribers of diverse backgrounds or geographical locations, even if the sheer number is not big, might be regarded as quite effective. In contrast, for a social media platform with a large number of subscribers, there could be many counts of feedback from only a small proportion of the total subscribers [15]. A better measure is closer to a ratio, which takes into account the number participation of users relative to a base. Also, older measures paid little attention to the interactions among users. When a game platform is implemented, it is useful to find out whether only a small or large cluster of users have interacted in the platform. In this conceptual paper, I seek to demonstrate how network measures can be used for these purposes.

## Methods

### A Possible Health Policy Simulation Game

#### Overview

Before discussing social network measures, it is useful to propose an actual game. Suppose a public health professor seeks to help students understand the ACA in the United States. While the ACA is relevant to a large group of stakeholders, in the game, players would also take a role. The role is supposed to represent a specific group of stakeholders, how these stakeholders may react to the ACA, and even how they negotiate and collaborate [16].

**Figure 1:**
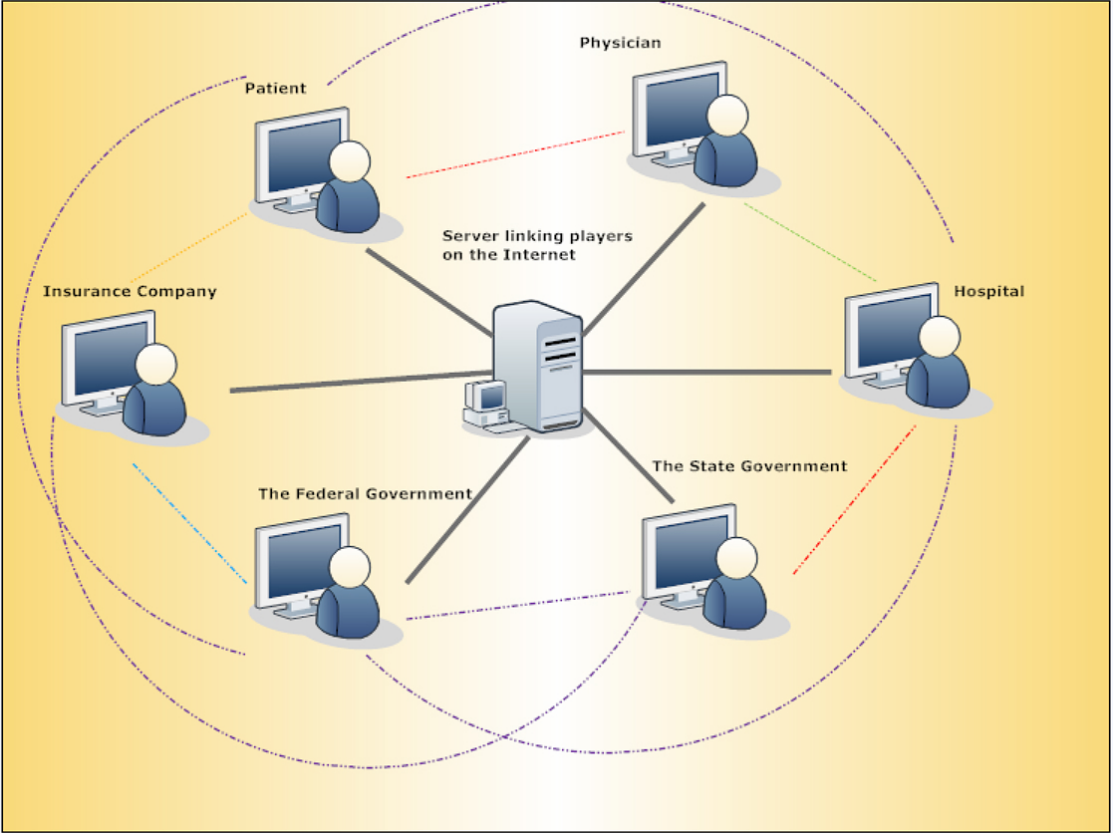
A Web-based simulation game of health policy.

For simplicity, I describe six possible roles: “the federal government”, “the state government”, “the hospital”, “the insurance company”, “the physician”, and “the patient”. These are the roles, but not necessarily the number of players. That is, each role may include multiple players. The game may be divided into different scenarios, or tasks, such as building the health insurance market place or expanding public health coverage in the ACA (Figure 1).

In the game, the player’s game performance would be assessed by different indicators. Briefly, players are motivated to play the chosen role successfully, like any other simulation game. Players would be required, and reminded regularly, to initiate communication and exchange information with other players (represented by the dotted lines in Figure 1). The game would require players to interact with other players and come up with joint actions. These interaction activities are of the main interest here, so they would be recorded by the backend computer, and analyzed by the researcher.

#### The Federal Government

In this role, the player may be given a hypothetical title of “ACA Secretary”, who is responsible for promoting the ACA to the general public, regulating and implementing the policy at the federal and state levels, and working with the academic and private sectors. In the simulated world, the player will take initiative to organize different activities, including meetings with researchers and business representatives, distributing research funding to study ACA policy implications, and so forth.

In the background, the game will generate random, but reasonable, environmental conditions that affect different stakeholders. How the game proceeds depends on these environmental conditions and actions are taken by different players. For example, once meetings with state-level government officials are done, the game can tell the federal government player how many attendees had a positive or negative reaction toward the new policy, how many states had decided to accept a specific federal government’s initiative, such as expanding the Medicaid, how many new research projects are completed with new funding, etc. Based on all these tasks, the game may compute a score about the player’s performance.

#### The State Government

In this role, the player may be given a hypothetical title of “State ACA Representative”. The player can choose his/her desired state with a certain orientation toward supporting or opposing the ACA due to politics, citizens’ local culture, and other state-specific characteristics. Like the federal counterpart, this role is also responsible for engaging the general public, and working with the academic and private sectors. But unlike the federal counterpart, this role is required to negotiate with the federal ACA secretary at various times, such as the autonomy the state can hold with regard to how state government agencies can use the new Medicaid funding.

Similar to the federal government role, the game will generate certain environmental conditions randomly in the background. For example, the game can tell the player how many attendees after a town hall meeting had a positive or negative reaction toward the ACA, how many still had confusion about the policy, and whether the state’s government officials have generally decided to support or oppose certain parts of the ACA. Again, based on various tasks, the player may be scored by the game at various times.

#### The Hospital

In this role, the player may be given the title of “Chief Executive Officer” (CEO). Like the state representative, the player can choose his/her desired locality with a certain business environment, political orientation, culture, and so on. Unlike the government roles, the hospital chief is more concerned with the policy’s outcomes, such as the organization’s relationship with patients and its business operation, than the policy’s implementation per se.

There will be different tasks to perform for this role as well. For example, as the ACA unfolds, the hospital officer will monitor how business revenues have or have not changed over time. The officer will also meet with other staff in the hospital periodically, including the Chief of Staff, the Chief Financial Officer, the Medical Director, and others to gauge their reaction toward the new policy. The CEO will also suggest new marketing policies, such as the use of social media.

In the background, the game will generate random environmental conditions, such as whether the hospital’s market share has been affected after the new law, or whether physicians have increased job satisfaction, among others. Based on various tasks, the player may be scored by the game at various times.

#### The Insurance Company

In this role, the player may be given the title of “Regional Director” of a hypothetical insurance corporation. Again, the player can choose his/her desired locality with a certain orientation toward supporting or opposing the ACA due to politics, culture, and so forth. The Regional Director is primarily interested in business performance by keeping a large enrollee base of the firm’s insurance packages.

The director’s everyday tasks, include negotiating with providers such as hospitals and physicians for discounted services, monitoring business performance, meeting with government officials to understand new policy initiatives, etc. The director may also develop new Health Maintenance Organization or Preferred Provider Organization packages to attract new enrollees.

In the background, the game will generate random, but reasonable, outcomes that affect the insurance company. For example, the game can tell the player whether the insurance company has received new analytical reports about a changing risk pool in the local population, whether the new law has affected profitability due to new mandatory requirements, whether providers are or are not willing to provide the desired discounts, among others. Based on various tasks, the player may be scored by the game at various times.

#### The Physician

In this role, the player can choose to become a solo-practice physician or one that is employed by a large hospital system. He/she can also choose to become a general practice or 1 of 24 specialties defined by the American Medical Association. Like the state representative, the player can choose his/her desired locality with a certain orientation toward supporting or opposing the ACA due to politics, culture, and so on. The physician will face different tasks and may encounter various job-related challenges. For example, he/she will need to meet with other staff in the health system periodically to discuss new reimbursement policies, penalties to hospitals for readmissions, patients’ engagement strategies, and others.

In the background, the game will generate random, but reasonable, outcomes that affect the physician’s work satisfaction, income level, relationships with colleagues and patients, and more. For example, the game can tell the player how the physician’s decision to adopt electronic medical records has increased the clinic’s efficiency and patients’ satisfaction. Based on the performance of various tasks, the player’s income will vary. The game can also assign an overall score to the player at various times.

#### The Patient

Finally, the player can assume the role of a patient in this game. Like other roles, the player can choose his/her desired locality with a certain orientation toward supporting or opposing the ACA due to politics or culture, among others. Since this role covers more variability in the actual population, the player may be given more choices in building the role. For example, the player can choose his income level, family status, health status, etc. For players who do not have a preference, the game may generate a given role status with specific profile variables. The player will manage the role based on these variables.

Subsequently, the patient would be interested in how the ACA can help them for various health-related tasks. These include whether they are eligible for Medicaid coverage under the new law, what types of insurance packages are available in the new Health Insurance Marketplace, what penalties they would face if they do not purchase insurance coverage, among others.

In the background, the game will generate random, but reasonable, outcomes that affect the patient in different ways. For example, the game can tell the player whether his or her health status has improved after the ACA, whether the family has obtained useful benefits, whether costs have been increased or decreased, and so on. Based on various tasks, the player may be scored by the game at various times.

### Evaluating the Game’s Utility

To evaluate collaboration, it is useful to think of players as representing stakeholders in the health sector. Stakeholders are members within a network, and stakeholders may be defined as individuals or groups who can affect or are affected by the achievement of certain organized objectives [17]. Early development of the stakeholder theory emphasized dyadic relationships between the organization and its stakeholders. This emphasis has been criticized as not paying sufficient attention to the interactions among stakeholders themselves [17].

In the context of simulation games, a focal organization (eg, a government agency or a college) may invite its members to play the game. The game essentially provides a network environment that enables its stakeholders to interact and build ties among themselves. These interactions can disseminate and produce useful knowledge.

### Network Density

**Figure 2:**
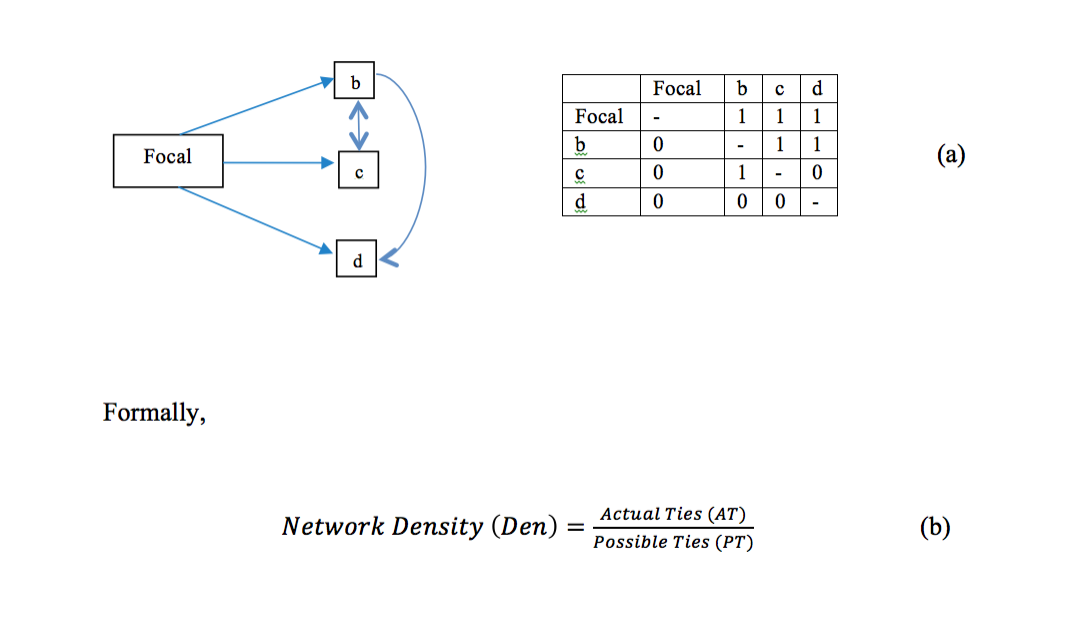
(a) Graph and matrix representations of stakeholder relationships, and (b) formal network density equation.

Whether the interactions are successful or not depends on how connected the game network is. The connectivity of a game network can be measured by a concept called “network density”. DEN indicates how fully the potential of connectivity is actually realized. For example, in Figure 2a, “Focal”, “b”, “c”, “d” are the players. With 4 players, the number of possible communication ties in the matrix of Figure 2a is 12 (ie, all the nondiagonal cells). If the number of actual ties is 6 (the cells with a “1”, indicating the presence of a communication exchange), DEN may be computed as 6/12 = 0.5.

It is noteworthy that one may distinguish between directional and nondirectional ties (ie, it does not matter whether an actor is a “sender” or “receiver” of the communication tie). For nondirectional ties (without “self-to-self” ties),

Possible Ties (PT) = (N) × (N-1)/2 (1)

where N=number of actors in the network. For example, when N=4, PT = 6 (the number of upper nondiagonal cells in a 4 × 4 matrix; Figure 2a). For directional ties (without self-to-self ties),

PT = (N) × (N-1) (2)

when N=4, PT = 12 (the number of all nondiagonal cells in a 4×4 matrix). If a researcher was only interested in whether a communication tie between 2 actors existed at all, then the use of a nondirectional tie is sufficient.

### The Whole is Greater than the Sum of Its Parts

**Figure 3:**
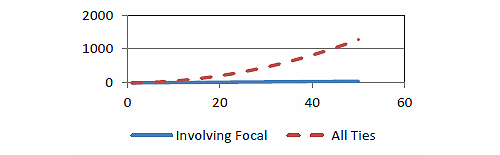
Comparison between the number of ties involving and not involving the focal organization.

Following Figure 2a, Web-based simulation games can generate more activities than the focal organization (eg, a school or a hospital) can directly involve. The engagement and the communication disseminates through peer-to-peer interactions. It is clear that the number of interactions increases exponentially relative to the increase in the number of stakeholders (Table 1 and Figure 3).

Using 10, 50, and 100 stakeholders (including the focal organization) as N for computation, in Figure 2, one can demonstrate the exponential increase, relative to the number of possible interaction ties that must involve the focal organization. Thus, in a network of N=10, the number of possible ties that must include the focal organization is 10; whereas the number of all possible ties in the same network (N=10) is 45. In a network of N=100, the corresponding figures are 100 vs 4950.

In other words, as the game enrolls more players, the influence increases exponentially. This provides a strong justification for enrolling a large number of players. These peer-to-peer exchanges may need meaningful collaborative activities (eg, development of new knowledge and social support groups). In this limited sense, building a social media platform (to enable interactions in the whole network) has a much greater value than summing up the dyadic ties between a focal organization and its stakeholders.

**Table 1:** Comparison between the numbers of ties involving and not involving the focal organization.

Number of stakeholders	Number of ties involving focal organization	Number of all ties
1	1	1
2	2	3
10	10	45
50	50	1225
100	100	4950

### Network Diversity

While DEN focuses on the existence (or nonexistence) of ties between actors in a network, connected actors may engage in repeated interactions (eg, back-and-forth communication exchanges). For example, in a game with 10 players (or N=10), 2 active players alone might have 10 repeated interactions. These were also the total number of interactions in the whole network. There were no exchanges in the rest of the game network (ie, everyone else remained silent). In another network of 10 stakeholders, there were also 10 interactions. But these interactions involved everyone in the network. Intuitively, the latter network was more “even”.

DIV quantifies this intuition of “evenness”. Assume again the focal organization has enrolled 9 players to its simulation game platform, creating a network of N=10. The interactions can be represented by a 10×10 matrix. Further assume that the researcher is only interested in nondirectional and non–self-to-self ties. This means that only the upper nondiagonal cells in the matrix need to be counted. Following Figure 2(b) the number of possible ties is (10) × (10-1)/2 = 45.

Yet, the game network allows repeated interactions between any pair of players. Thus, the value in each cell of the matrix could have a value other than 1 and 0. For example, between actors 1 and 2, there could be two, three, four, or any positive number of communication exchanges. DIV considers whether all interactions within a network are concentrated among a relatively small group of actors or the interactions are evenly distributed among all actors.

In the context of simulation games, the questions are: Do interaction exchanges concentrate among a small group of active players? Or do interaction exchanges take place between all enrolled players?

**Figure 4:**
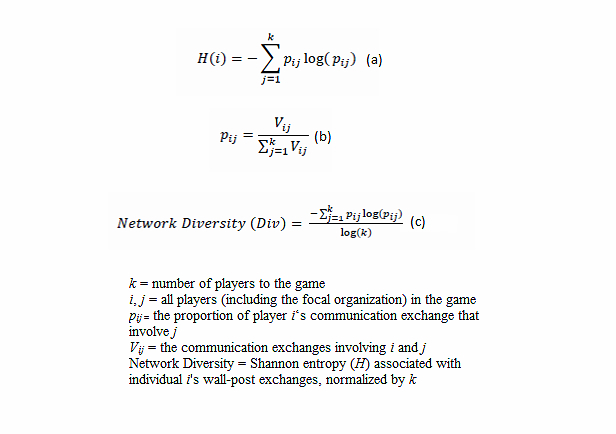
(a) Represents the Shannon’s diversity index equation. (b) Represents the communication exchange between the players. (c) Shows network diversity as it relates to Shannon's entropy and player's wall postings.

To define DIV, I borrow the concepts of topological diversity and Shannon entropy from ecological research. The major formulae from the concepts enable me to measure the number of activities within a network in conjunction with the network’s size. Figure 4 describes how DIV is derived.

To interpret this measure (DIV), assume that an online game was initiated by a hospital to introduce a new health policy. A high DIV score suggests that communication exchanges are evenly distributed among all enrolled players, so the dissemination value of the game may be regarded as relatively high; a low score suggests that communication exchanges are concentrated among a small group of players, so the dissemination value may be considered relatively low.

It is also useful to reveal whether certain roles of players tend to collaborate more often in specific policy scenarios. For example, when building the health insurance market place, do the roles of insurance companies and the state government collaborate more frequently?

Both measures capture collaborations at a specific time (ie, cross-sectional). Researchers need repeated application of these measures to capture changes and stability over time (ie, longitudinal).

## Results

Suppose a hypothetical game on the ACA is built (Figure 3), how can researchers use the 2 network measures to evaluate its utility? I recommend the following steps in Textbox 1.

With data collected in step 4 of Textbox 1, the researcher is able to evaluate the collaboration utility of the game based on the 2 measures discussed above. For example, the researcher may examine how DEN increases or decreases after the game started. With sufficient data, the researcher can even come up with estimates regarding how soon online interactions would die down. This would enable the organizer to adjust the game’s time span in step 6 shown in Textbox 1.

Similarly, the researcher may use DIV to analyze where the interactions are most frequent. For example, do interactions tend to come from players who assume certain roles (eg, physicians and patients)? Would interactions generate different clusters, such as clusters of physicians and patients and clusters of federal and state governments?

Finally, if the researcher is able to solicit additional participation from players, it is possible to examine whether or not, and how, social networks augment the individual benefits that players obtain from the game. Table 2 includes several research questions that are made possible with the 2 social network measures.

**Steps to evaluate the utility of network measures.**

1. Specify the pedagogical goals of the simulation games,
2. Develop scenarios to segment the game in line with the pedagogical goals,
3. Put the games on Web-based platforms (preferably with attractive graphics) that can generate random and reasonable environmental conditions,
4. The background computer should be able to record interactions among players and perform social network analysis as specified in the previous section,
5. Nominate a credible organizer (eg, an educational institute) who would take the responsibility of initiating the game (even though the analysis of the above measures is handled by the computer),
6. Recruit players to participate in the game. Preferably the number of players for each role is even in the game,
7. Determine an official start and end times for the game (or for each scenario),
8. Appoint a researcher to evaluate the utility of the game,
9. Organize one or more postgame session(s) to debrief participants with research findings from the researcher, and
10. Consider modifying the game and/or restarting the game with another group.

**Table 2:** Knowledge- and affection-based benefits of online games augmented by network density and diversity.

	Knowledge-based	Affection-based
DEN	Whether many players have acquired knowledge about health policy within networks?	Whether many players have shown increased support to a health policy within networks?
DIV	Whether players interact evenly within health networks regarding health policy?	Whether players influence one another evenly within networks regarding health policy?

## Discussion

In the health sector, networks continue to be important in linking organizations and individuals together. Web-based simulation games offer an opportunity to build social networks linking different stakeholders. As I attempt to show in this paper, the focal organization might still play the “broker” role to organize a game to increase legitimacy of playing. Yet, it is the actual interactions among individual players that matter. As my analysis shows, the greater the number of players, the greater the potential for stakeholder engagement, relative to the direct influence exerted by the focal organization.

On the other hand, the above measures only evaluate the effectiveness of social networking in games structurally. The contents covered in the game, such as what and how to play the game, need to be studied in other innovative ways. The content in the online interactions among players is important as well. One fruitful research direction is to collect and analyze empirical data from actual online game networks. Researchers can then reconsider whether the interpretations above make practical sense.

Given the availability of existing computer programs, software and application programming interface, analyzing empirical data with the network measures discussed is quite viable at the implementation level. One possible approach is to expand existing mobile apps and/or games developed by commercial vendors with algorithms that compute the social network measures discussed in this paper. This approach will speed up the process of developing a real product to be used in the health sector. Further research should also consider the impact of games in different geographical areas. For example, researchers have found that video games are extremely popular in East Asian countries, such as South Korea and China [18]. Will the social networks potential of games be high in these countries? What are the constraints and opportunities to use online games for health promotion purposes in these countries? I believe future research publications and professional conferences should address these meaningful questions.

## Abbreviations

CEO: chief executive officer
DEN: network density
DIV: network diversity
PPACA or ACA: Patient Protection and Affordable Care Act
PT: possible ties
